# Unilateral pleural effusion with capillary haemangioma

**DOI:** 10.1002/rcr2.613

**Published:** 2020-06-25

**Authors:** Nozomi Kadota, Manabu Murakami, Ryosuke Imai, Torahiko Jinta, Tomohide Tamura

**Affiliations:** ^1^ Department of Pulmonary Medicine, Thoracic Center St. Luke's International Hospital Tokyo 104‐8560 Japan

**Keywords:** Benign vascular tumour, haemangioma, mediastinal tumour, pericardial tumour, unilateral pleural effusion

## Abstract

Here, we report a case of haemangioma on middle mediastinum accompanied by unilateral pleural effusion, which was initially suspected to be lung cancer and pleurisy. During annual check‐up, chest radiography of a 30‐year‐old female showed homogeneous opacity in the left lower pulmonary field. Excision was performed, and the mass was pathologically diagnosed as benign mediastinal vascular tumour with exudative pleural effusion. To our knowledge, this presentation occurs in <0.5% of tumours of the mediastinum, and furthermore, the presence of pleural fluid is extremely rare, and the underlying mechanism is unknown. Although mediastinal haemangioma is hard to diagnose without surgery, we should include it in the differential diagnosis of a tumour with unilateral pleural effusion.

## Graphical abstract

Here, we report a case of haemangioma on middle mediastinum accompanied by unilateral pleural effusion, which was initially suspected to be lung cancer and pleurisy. Although mediastinal haemangioma is hard to diagnose without surgery, we should include it in the differential diagnosis of a tumour with unilateral pleural effusion.

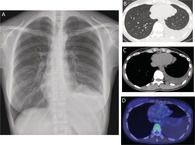

## Introduction

Mediastinal haemangioma is a very rare neoplasm, present in <0.5% of mediastinal masses. Here, we report a rare case of a mediastinal mass accompanied by unilateral pleural effusion impossible to be diagnosed without excision.

## Case Report

A 30‐year‐old non‐smoker female was referred to our outpatient clinic with left‐sided limited pleural effusion on X‐ray radiography, detected at an annual health check‐up. She had a past medical history of endometriosis, ovarian cyst, and fibroadenomas of the left breast, which were all treated two years ago by polypectomy of the uterine cervix and hormone therapy. For the past eight years, she has been travelling abroad once a month for business, from east Asia to Europe, and had been to a coal mine in Indonesia for more than 15 times. She had been followed up since the last eight years, with no definitive diagnosis; moreover, she had no cough, sputum, or breathing difficulty.

At our clinic, the patient appeared generally good and conscious, with a body temperature of 37.1°C, blood pressure of 108/70 mmHg, and heart rate of 70 beats/min. Her respiratory rate was 16 cycles/min and oxygen saturation was 96% on room air. Cardiopulmonary examination revealed diminished breath sounds on the left lower lung lobe and dullness on percussion was also noted. All other findings on physical examination were unremarkable. Laboratory tests showed normal blood levels (leucocytes: 5500/μL; neutrophils: 3380/μL, eosinophils: 200/μL, lymphocytes: 1480/μL, and monocytes: 390/μL), C‐reactive protein (<0.04 mg/dL), interleukin‐2 receptor (293 U/mL), and tumour markers (carcinoembryonic antigen (CEA): 0.5 ng/mL, stage‐specific embryonic antigen‐1; (SSEA‐1) sialyl Lewis X‐i antigen (SLX): 34.9 U/mL, carbohydrate antigen 19‐9 (CA 19‐9): 7.9 U/mL).

**Figure 1:**
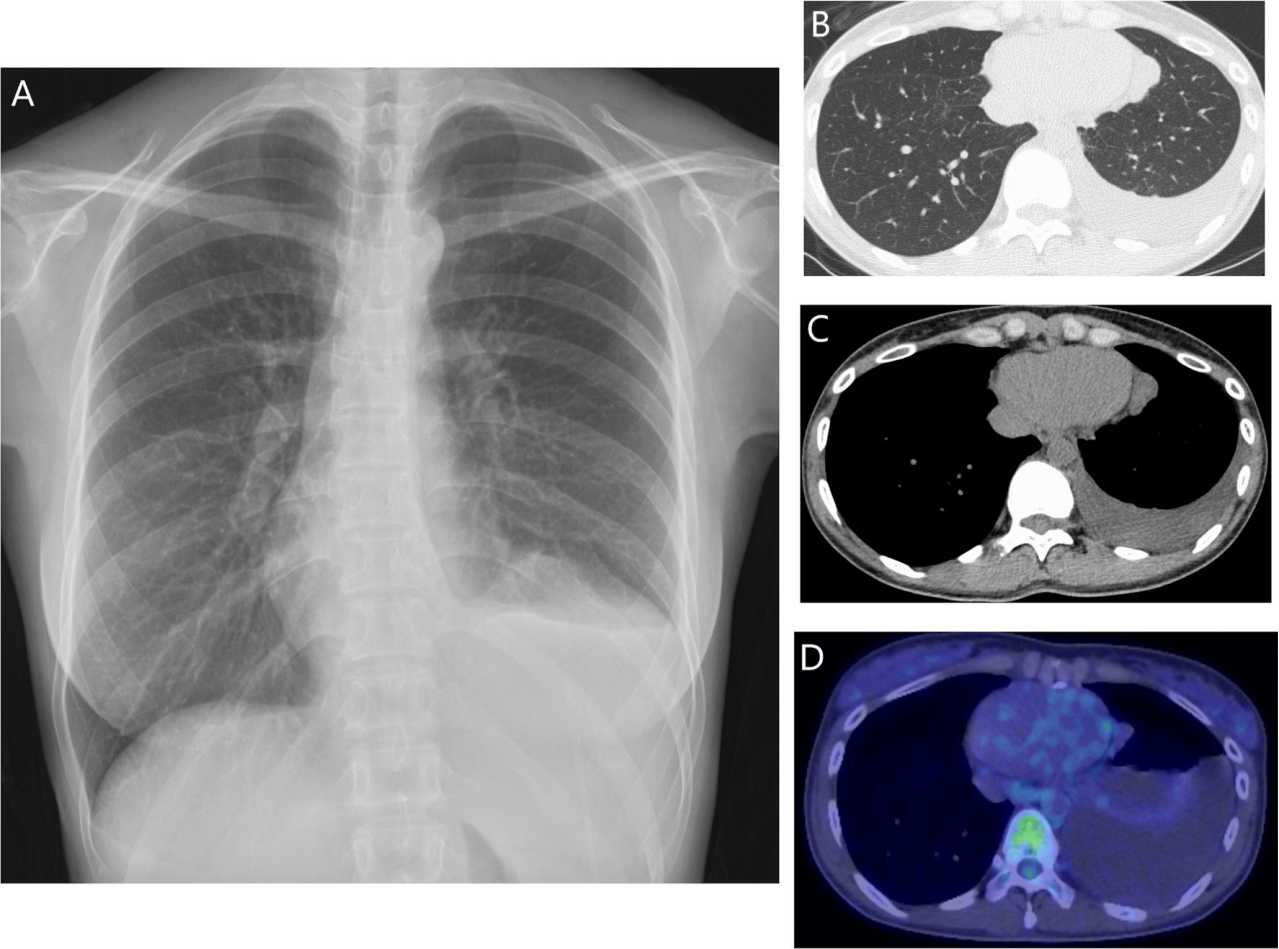
(A) Chest radiography showing a homogeneous opacity in the left lower pulmonary field. (B, C) High‐resolution computed tomography (CT) showing scattered multiple mass lesions on the medial side of the left lower lobe. (D) Axial chest positron emission tomography–CT (PET–CT) scan showing no 18F‐fluorodeoxyglucose (FDG) uptake. However, it shows increased pleural effusion.

Chest radiography showed a homogeneous opacity in the left lower pulmonary field. On high‐resolution computed tomography (HRCT), multiple mass lesions on the medial side of the left lower lobe appeared to be scattered, which are supposed to be pleural in origin. They showed strong and non‐uniform contrast enhancement, with the largest one being 27 × 15 × 33 mm, which appeared to be nourished by the musculophrenic branch of the left internal thoracic artery (Fig. 1). Also, there was a lesion of about 15 mm in diameter at the right anterior segment of the liver. It showed hypoattenuation on non‐contrast images and peripheral enhancement on contrast study, which is most likely representative of hepatic haemangioma. Pleural mesothelioma, pseudomesothelioma, lung cancer, other sarcomas, and pleural metastases were considered as differential diagnoses, although there was no significant mediastinal/hilar lymphadenopathy. Left‐sided diagnostic thoracentesis was performed, and the aspirated pleural fluid, of light yellow colour, had 162 cells/μL, with 10% lymphocytes and 8.5% neutrophils, and adenosine deaminase (ADA) was 4.9 U/L. As the ratio of effusion protein/serum protein was 0.6 (4.3/7.1) and its effusion lactate dehydrogenase (LDH)/serum LDH 0.5 (68/134), the fluid was slightly exudative. Pleural fluid cultures revealed no bacteria or mycobacteria, and no malignant cells were detected on cytological examination of the pleural fluid.

**Figure 2:**
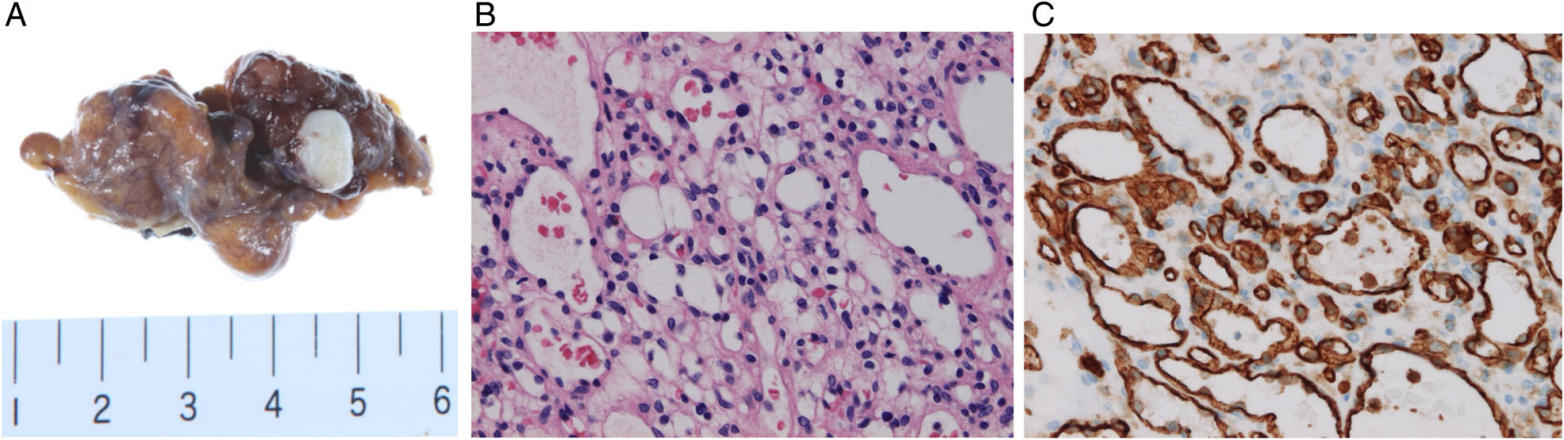
(A) Video‐assisted thoracoscopic surgery demonstrating the appearance of the tumour. Haematoxylin–eosin staining (B) and immunostaining (C) demonstrating the diagnosis of capillary haemangioma of the mediastinum.

A week later, positron emission tomography–computed tomography (PET–CT) was performed and indicated no 18F‐fluorodeoxyglucose (FDG) uptake, and the pleural effusion had increased (Fig. 2). After a multidisciplinary meeting among respiratory medicine doctors, thoracic surgeons, and radiologists, we chose a way to make a definitive diagnosis at an early stage to exclude a malignant tumour for a previously healthy young woman, even though there was no symptom nor PET accumulation. Neither CT‐guided biopsy nor Endobronchial ultrasound‐guided transbronchial needle aspiration (EBUS‐TBNA) could be performed anatomically, so video‐assisted thoracoscopic surgery (VATS), thoracoscopic tumorectomy, and pleural biopsy were performed for diagnosis. The tumour surface was smooth with a covering of white pleura, and it was elastic soft. Both pleural biopsy and partial resection of upper lobe of left lung showed negative results, except for mild inflammatory changes. Vascular endothelial cells, positive for anti‐CD31 antibody, were strongly positive, and CD99, which implies solitary fibrous tumour, was negative. Finally, the diagnosis of capillary haemangioma of the mediastinum was made pathologically. On contrast CT after four months of the operation, there is no recurrence of fluid nor tumour.

## Discussion

Pleural effusions are a common medical problem with over 50 recognized causes [1]. Mediastinal capillary haemangioma is extremely rare, especially of the middle mediastinum, and accounts for <0.5% of mediastinal masses [2]. The challenging point for this rare tumour is determining a preoperative diagnosis as no specific diagnostic imaging hallmarks have been reported. The first case of a benign mediastinal haemangioma was reported by Shennan in 1914 [3]. The age of affected patients is wide (three months to 76 years) and more than half were younger than 35 years at diagnosis. There is no sex predilection [4]. In this case, if we consider the patient's medical history, the hormone therapy for endometriosis and ovarian cyst could have been the cause of the hepatic haemangioma [5]. However, there is neither a known relationship between hormone therapy and formation of mediastinal haemangioma, nor an association with fluid effusion. Since little thoracic effusion remained after a month, we inferred that the fluid was formed by the tumour and should not recur. In conclusion, we report a case of benign vascular tumour of mediastinum with one‐sided pleural fluid, which was suspected to be lung cancer. Although surgery could not be avoided for diagnosis, we should include mediastinal haemangioma in the differential diagnosis of a tumour with pleural fluid.

### Disclosure Statement

Appropriate written informed consent was obtained for publication of this case report and accompanying images.
